# Editorial for Special Issue “Plant Genetics and Molecular Breeding”

**DOI:** 10.3390/ijms20112659

**Published:** 2019-05-30

**Authors:** Pedro Martínez-Gómez

**Affiliations:** Department of Plant Breeding, CEBAS-CSIC, P.O. Box 164, 30100 Espinardo, Murcia, Spain; pmartinez@cebas.csic.es; Tel.: +34-968-396-200

The development of new plant varieties is a long and tedious process involving the generation of large seedling populations to select the best individuals. In addition, the requirements for new varieties must be anticipated several years in advance (between five in the case of horticultural crops and 15 in the case of fruit crops), depending on the average time from the original cross to the release of a new pre-crop variety. One important decision is the choice of which parents to use. Subsequent crosses can include the complementary type (the cross of two varieties with complementary characteristics to obtain a new variety that integrates the positive aptitudes of both varieties) or the transgressive type, in which two varieties are crossed with positive aptitudes to obtain progeny performance that supersedes either parent [1]. Although the ability of breeders to generate large populations is almost unlimited, the management, phenotyping (genetic studies), and selection of seedlings are the main factors limiting the generation of new cultivars [2]. In this context, molecular (DNA) studies for the development of marker-assisted selection (MAS) strategies are particularly useful when the evaluation of character is expensive, time-consuming, or comprises long juvenile periods. In addition, proteomic (proteins and enzymes), transcriptomic (RNA), and epigenetic (DNA methylation and histone modifications) studies are being applied to breeding programs [3]. 

Integrated approaches have been classically performed for the genetic characterization of plant material [4]. However, the current development of suitable markers applicable in the selection for agronomical traits must be used for the clarification of the above-mentioned genomic studies and the development of more efficient markers. In this context, this Special Issue, “Plant Genetics and Molecular Breeding”, presents a total of 34 articles with 33 original research articles and one review article broadly covering the field of genetics and molecular plant breeding from a multidisciplinary perspective (Table 1). Manuscripts focus on the integration of different phenotypical and molecular tools for the analysis of different traits with a high interest in breeding. These multidisciplinary studies have been performed predominantly in cereal crops including rice [5,6,7,8,9,10,11,12,13] and wheat [14]; industrial crops including oilseed rape [15,16,17], sugarcane [18,19], soybean [20], sesame [21], and minor oil crops [22]; vegetable crops including cabbage [23,24], tomato [25], broccoli [26], chickpea [27], and cucumber [28]; ornamental crops including *Chrysanthemum* [29,30], *Paeomia* [31], rose [32] and *Aechmea* [33]; fruit and nut trees such as kiwi [34] and almond [35]; and crops with environmental [36,37] and medical uses [38].

Papers published in this Special Issue has reported high novelty results as well as plausible and testable new models for the integrative analysis of the different approaches applicable to plant breeding, including genetic (phenotyping and transmission of agronomic characters), physiology (flowering, ripening, organ development), genomic (DNA regions responsible for different agronomic characters), transcriptomic (gene expression analysis of the characters), proteomic (proteins and enzymes involved in the expression of the characters), metabolomic (secondary metabolites) and epigenetic (DNA methylation and histone modifications) approaches (Table 1). The objective of these studies is the development of new MAS strategies linked to the most important agronomic traits. In this context, these integrated approaches have been applied to the analysis of different agronomic traits with a focus on breeding related to an increase in production (nutrient use efficiency, yield, pollen development, plant development, cytoplasmic male sterility, elongated internode, abortive buds), increase in quality (grain quality, starch composition, phenolic acids, leaf colour, flower colour, polyunsaturated fatty acids, plant architecture, flower development, floral scent), and the reduction of costs with biotic (nematode resistance) and abiotic (flowering time, drought resistance, waterlogging resistance, salt stress, heat tolerance, heavy metal tolerance) stress tolerance (Table 1). 

Overall, the 34 contributions published in this Special Issue (Table 1) illustrate the advances in the field of plant genetics and molecular breeding as well as the different integrated approaches necessary for plant breeding programs of the 21st century. The application of massive sequencing methodologies (“deep-sequencing”) of the genome (DNA-Seq) [14,17,21], transcriptome (RNA-Seq) [15,19,20,28,37], and proteome [23], focused on lowering the costs of sequencing technologies, has been also widely reported in this Special Issue. These methodologies allow for broader knowledge of the complete genome and transcriptome, respectively. Currently, this application is of great interest to breeding programs, considering the high number of plants species with reference genomes [39]. 

To conclude, we assert that human activities are producing a significant increase in global temperatures, a phenomenon referred to as climate change. According to the “Intergovernmental Panel on Climate Change (IPCC) Fourth Assessment Report”, the average global temperature has increased by 0.74 °C over the last century and is expected to rise between 1.1 °C and 6.0 °C before 2100 [40]. Climate change is affecting all life processes on earth, including food crop production. Increases in temperature are modifying the growth stages of plants, especially those in temperate zones that are adapted to seasonal changes in solar radiation, temperature, and water availability. These molecular approaches at genomic, transcriptomic, proteomic, metabolomic and epigenetic levels, together with an increasingly accurate phenotyping, will facilitate the breeding of new climate-resilient varieties resistant to abiotic stress with a suitable productivity and quality to extend the adaptation and viability of current varieties. 

## Figures and Tables

**Table 1:** Contributors to the Special Issue “Plant Genetics and Molecular Breeding”.

Crop	Species	Trait	Integrated Approaches	Reference
**Cereal**	Rice	Nutrient use efficiency	Physiology, Genomics	Jewel et al. [5]
			Physiology, Genomics, Transcriptomics	Ali et al. [6]
		Grain yield	Genomics, Transcriptomics, Transformation	Fu et al. [7]
			Genetics, Genomics, Transcriptomics,	Zhang et al. [8]
		Starch accumulation	Physiology, Genomics, Transcriptomics	Zha et al. [9]
		Starch composition	Genomics, Transcriptomics, Transformation	Jiang et al. [10]
		Pollen development	Physiology, Genomics, Transcriptomics	Sun et al. [11]
		Endorpesm development	Genomics, Transcriptomics, Transformation	Wang et al. [12]
		Plant development	Genomics, Transcriptomics, Transformation	Xue et al. [13]
	Wheat	Drought stress	Physiology, Genomics	Bhatta et al. [14]
**Industrial**	Oilseed rape	Cytoplasmic male esterility	Physiology, Genomics, Transcriptomics	Ding et al. [15]
		Nematode resistance	Genomics, Transcriptomics, Transformation	Zhong et al. [16]
		Heavy metal tolerance	Genomics, Transcriptomics, Metabolomics	Pan et al. [17]
	Sugarcane	Stem borer resistance	Physiology, Genomics, Transformation	Gao et al. [18]
		Plant growth	Genomics, Transcriptomics, Genetic Transformation	Wang et al. [19]
	Soybean	Branching	Physiology, Genomics, Transcriptomics	Shim et al. [20]
	Sesame	Yield	Genetics, Genomics, Transcriptomics	Zhou et al. [21]
	Oil crops	Polyunsaturated fatty acids	Physiology, Genomics, Transcriptomics	Wu et al. [22]
**Vegetable**	Cabbage	Cytoplasmic male sterility	Genomics, Transcriptomics, Proteomics	Han et al. [23]
		Leaf colour	Genetics, Genomics, Transcriptomics	Liu et al. [24]
	Tomato	Elongated internode	Genomics, Transcriptomics, Transformation	Sun et al. [25]
	Broccoli	Abortive buds	Genetics, Genomics, Transcriptomics,	Shu et al. [26]
	Chickpea	Heat tolerance	Genetics, Genomics, Transcriptomics	Paul et al. [27]
	Cucumber	Drought stress	Physiology, Genomics, Transcriptomics	Wang et al. [28]
**Ornamental**	*Chysanthemun*	Flower development	Physiology, Genomics, Transcriptomics	Yang et al. [29]
		Salt stress	Genomics, Transcriptomics, Transformation	He et al. [30]
	*Paeonia*	Flower colour	Genomics, Transcriptomics, Transformation	Zhang et al. [31]
	Rose	Flower coluor	Genomics, Transcriptomics, Transformation	Sui et al. [32]
	*Aechmea*	Plant architecture	Genomics, Transcriptomics, Transformation	Lei et al. [33]
**Fruit tree**	Kiwi fruit	Waterlogging resistance	Physiology, Genomics, Transcriptomics	Pan et al. [34]
	Almond	Flowering time	Physiology, Genomics, Epigenetics	Prudencio et al. [35]
**Environmental**	Desert moss	Drought tolerance	Genomics, Transcriptomics, Transformation	Li et al. [36]
	Wintersweet	Floral scent	Physiology, Genomics, Transcriptomics,	Li et al. [37]
**Medical**	*Salvia*	Phenolic acids	Transcriptomics, Transformation, Metabolomics	Wang et al. [38]

## Abbreviations

DNA-Seq	DNA sequencing
MAS	Marker-assisted selection
RNA-Seq	RNA sequencing
